# Evaluation and analysis of image compression effect on neural network-based heart rate classification

**DOI:** 10.1038/s41598-025-06031-8

**Published:** 2025-07-02

**Authors:** Tianyu Dong, Seongho Cook, Jaiyoung Oh, Euee S. Jang

**Affiliations:** 1 Department of Computer Science, Hanyang University, 222 Wangsimni-ro, Seongdong-gu, Seoul, 04763 Republic of Korea; 2 Theragen Bio, Seongnam-si, 13488 Gyeonggi-do Republic of Korea

**Keywords:** Heart rate, Image compression, Neural network, Classification, Data processing, Image processing, Machine learning

## Abstract

In this study, we evaluated and analyzed the effects of image compression on a neural network (NN)-based heart rate (HR) classification system. An NN-based HR-estimation system classifies facial images into groups of HR intervals. We evaluated the relationship between the image compression rates and accuracy of an NN-based HR estimation system. In our evaluation, the image of the face was compressed into lossless (PNG) and lossy (JPEG) formats to reduce the transmission bandwidth. The compressed images significantly reduce the required bandwidth and storage size. Furthermore, we analyzed the image classification accuracy of the DenseNet-121, VGG-16, and Inception V3 models. VGG-16 exhibited the highest performance, and the proposed system yielded an accuracy of 97.2% for correctly detecting the HR. Additionally, the results showed that lossy image compression quality slightly affected HR accuracy. This evaluation method can provide an effective solution under low computational complexity and low bitrate requirement for remote HR classification.

## Introduction

In recent years, several studies have been conducted on personal health-related numerical recording and monitoring technologies. Heart rate (HR) has also been used in various applications such as the measurement of fitness exercise or diagnosis of cardiovascular disease. When measuring HR of a target human, additional hardware such as a photoplethysmogram (PPG), an electrocardiography (ECG) sensor is used. Although these wearable devices can help achieve higher accuracy with the help of neural networks^1,2^, the devices that must be worn directly on the body may cause an uncomfortable experience for the monitored subject. Wired sensors are not appropriate for subjects performing strenuous exercises, and wearable devices must be attached to the subject at a proper position.

To overcome the disadvantages of contact HR measurement methods, some studies have recently been conducted to estimate HR using visual methods, such as video sequences, or using the camera sensor of a portable device directly^3^. Therefore, the HR can be estimated by detecting color changes on the face caused by heart-generated pulse waves. Many researchers have focused on a region of interest (ROI) on the forehead or mouth that is rich in capillaries. In addition, filtering techniques are used to reduce the noise caused by ambient light and motion of the target human^4^. Moreover, acquiring HR using a high-performance smartphone camera is possible because it is easier to handle while exercising.

However, removing the effects of noise from environmental light and the motion of the subject remains a challenging task. Some recent studies have employed an NN to estimate the HR. A two-step convolutional neural network (CNN)-based method has been proposed to obtain results superior to those of the three standard methods^5^.

In these studies, a long video sequence was used as input data. Transferring and storing video data is highly challenging owing to the required storage size. For example, using MPEG AVC to compress a 1080p resolution video (Level 4 profile) requires a bandwidth of 50 Mbits for transfer^6^. Instead of delivering the full video sequence, classifying the key frame of the sequence and cropping out the facial part of the target human would significantly reduce the data size. An image-compression technique can also be used in the transmission process to reduce the required bandwidth.

In this study, we evaluated a simplified design using an NN-based image classification system and its performance using compressed images. The proposed design uses image files as inputs and estimates the HR interval using an NN-based image classification. Additionally, we evaluated the effect of compressed images on performance.

The remainder of this paper is organized as follows. In “Background and related work”, the background of the existing HR estimation technology is reviewed. A detailed procedure for the implementation and evaluation process is presented in “Proposed method”. In “Experiment result”, the training procedure and the evaluation results are discussed. We draw our conclusions in “Conclusion”.

## Background and related work

### Heart rate estimation

**Fig. 1:**
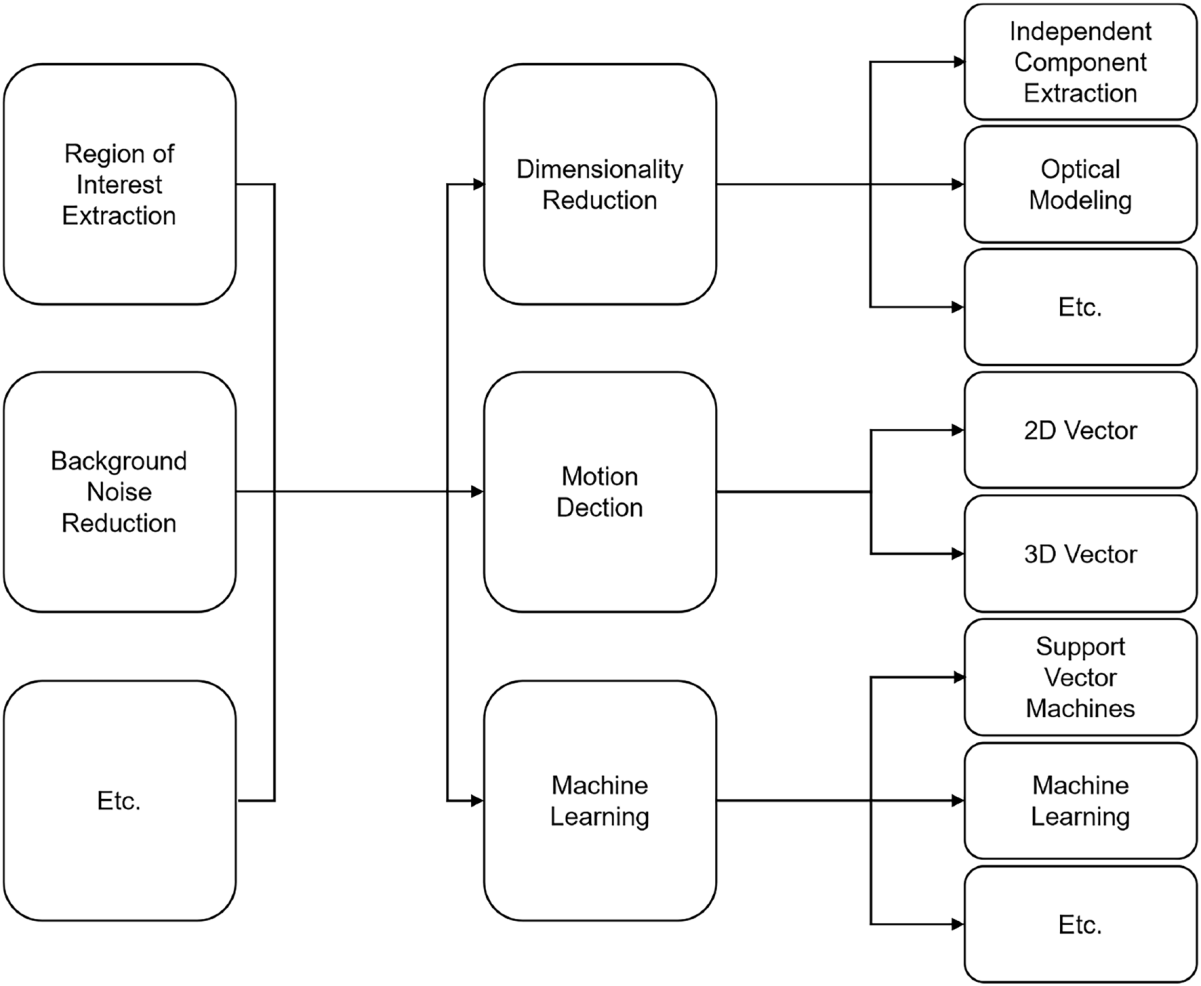
Summary of the remote HR estimation.

The main categories of HR detection are heart sound analysis, PPG, and ECG^7,8^. These methods have been used to detect the HR, which requires contact sensors or a microphone to be fixed on the test subject. However, to overcome the limitations of contact-based techniques, recent studies have focused on remote HR estimation systems. Most remote estimating systems can be categorized into dimensionality reduction, motion detection, and machine learning-based methods^9^, as shown in Figure 1.

These HR estimation methods use images as the input source data. Background noise reduction and region of interest (ROI) are common preprocessing utilities used to enhance results. Background noise reduction can decrease the effect of ambient light on test subjects. The ROI detection attempts to select the area that was most affected by the pulse of the test subjects. Dimensionality reduction is a broad category for analyzing pulse signals in a video. Investigations based on independent and principal component analyses aim to simplify the relationship between cardiac information and the RGB color space^10^. Another category is optical modeling^11^, which determines the changes in intensity caused by a pulse. In recent studies, a trending research direction has been the exploration of the green channel in RGB and chrominance in YCrCb color spaces, owing to their sensitivity to pulse-induced variations. Motion detection-based methods attempt to identify micromotions caused by carotid artery blood pressure. Two-dimensional^12^ and three-dimensional (3D)^13^ motions have been used in some studies to estimate HR. Furthermore, machine learning-based methods have also been employed to estimate HR. Support vector machines or neural networks enhance the prediction of HR, which may result in lower error compared to ground-truth method^5,14^. However, all of these methods are based on continuous datasets of image sequences and cardiac information.

### Image compression

The datasets used in previous studies were recorded in the format of video files. More recent datasets have been recorded with a high pixel definition, leading to an increase in the size of video files. For example, one minute of uncompressed video with a 1920 $\times$ 1080 resolution requires a storage capacity of 177 MB. Some of the accessible datasets are listed in Table 1. It should be noted that the video size of the test subjects was quite large.

**Table 1:** Example of public datasets used for the HR estimation.

Dataset properties	Datasets		
	CohFace	Pure	Ecg_fitness
Total size	300 MB	41 GB	1 TB
Video sequence Quantity	160	59	98
Resolution	640$\times$480	640$\times$480	1920$\times$1080
Compression method	MPEG-4	PNG	Raw
Size per sequence (MB)	$\sim$2	$\sim$680	$\sim$5300

Even if these videos can be compressed using different compression methods, the total size would be large for many subjects. Consequently, detection methods that rely only on facial parts are needed.

## Proposed method

### Design of the classification system

**Fig. 2:**
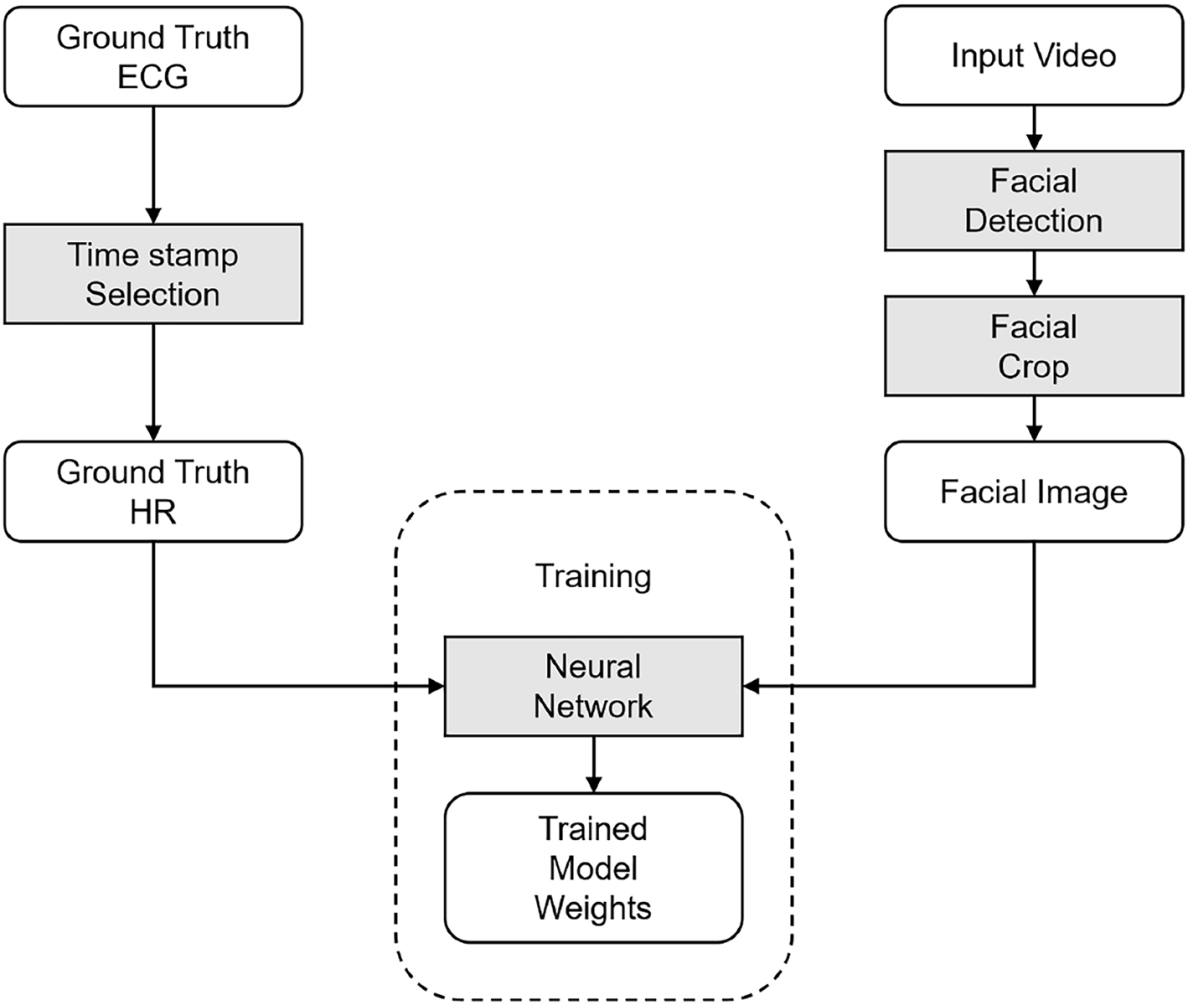
Details of the proposed training system.

**Fig. 3:**
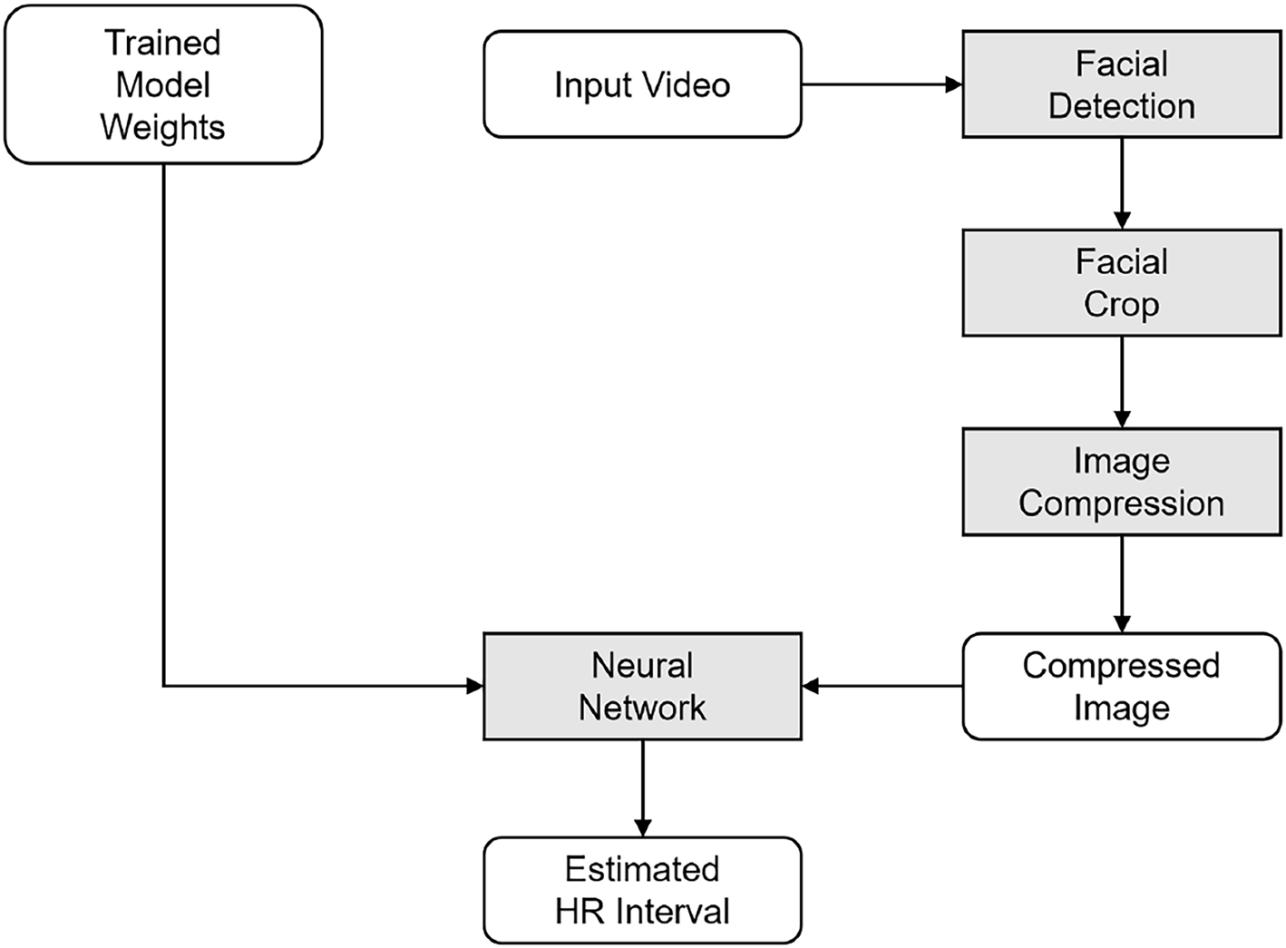
Details of the proposed inference system.

Figures 2 and 3 illustrate the proposed system, including the input data preprocessing, training, and evaluation. In Fig. 2, the input data preprocessing comprises facial detection and timestamp matching. Subsequently, the training process was applied to the selected network. Finally, the trained models were tested on images of different quality, as shown in Fig. 3. To reduce the size of the input data and focus on the facial part of an image, facial detection and data preparation were executed. The selected facial detection tool generated a set of 256 $\times$ 256 images from the captured video. These images were stored in compressed image files for further processing. The images were classified into different labels with the nearest corresponding ground truth HR, based on the timestamp. In the second step, the preprocessed images are separated into training, evaluation, and test sets. The training and evaluation sets used lossless compression, whereas lossy compression was applied to the testing set.

The lossless compressed training dataset is fed into the training process. Each image was entered separately as input into the network. After training, a model file was generated for each network, which could be used in the evaluation process. The evaluation considered the prediction precision and different qualities of the compressed images fed into the inference process. The statistical results were recorded when the images were correctly classified.

### Implementation of the system

To implement the proposed system, we select three networks based on their image classification performance: DenseNet-121^15^, VGG-16^16^, and Inception V3^17^.

We used a tool for the facial crop based on SeetaFace2, FFmpeg, and libjpeg-turbo^18–20^. The SeetaFace2 module recognizes the face and provides coordinates on the video. Subsequently, we used FFmpeg to crop a 256 $\times$ 256 image from the provided coordinates and saved it into an uncompressed raw video format to avoid any distortion between the color space conversions. We chose a lossless PNG to store images for the training and evaluation processes. Libjpeg-turbo, an unofficial reference software, was used to compress the test data. Different quality factors were used to generate different levels of compressed test data. The cropped images were labeled with HR intervals. In addition, we labeled each image with its nearest timestamp, corresponding to the HR data. The data were randomly assigned to one of the training, validation, or test sets. The ratio of the training, validation, and test sets was 70:15:15.

## Experiment result

### Data set

**Fig. 4:**
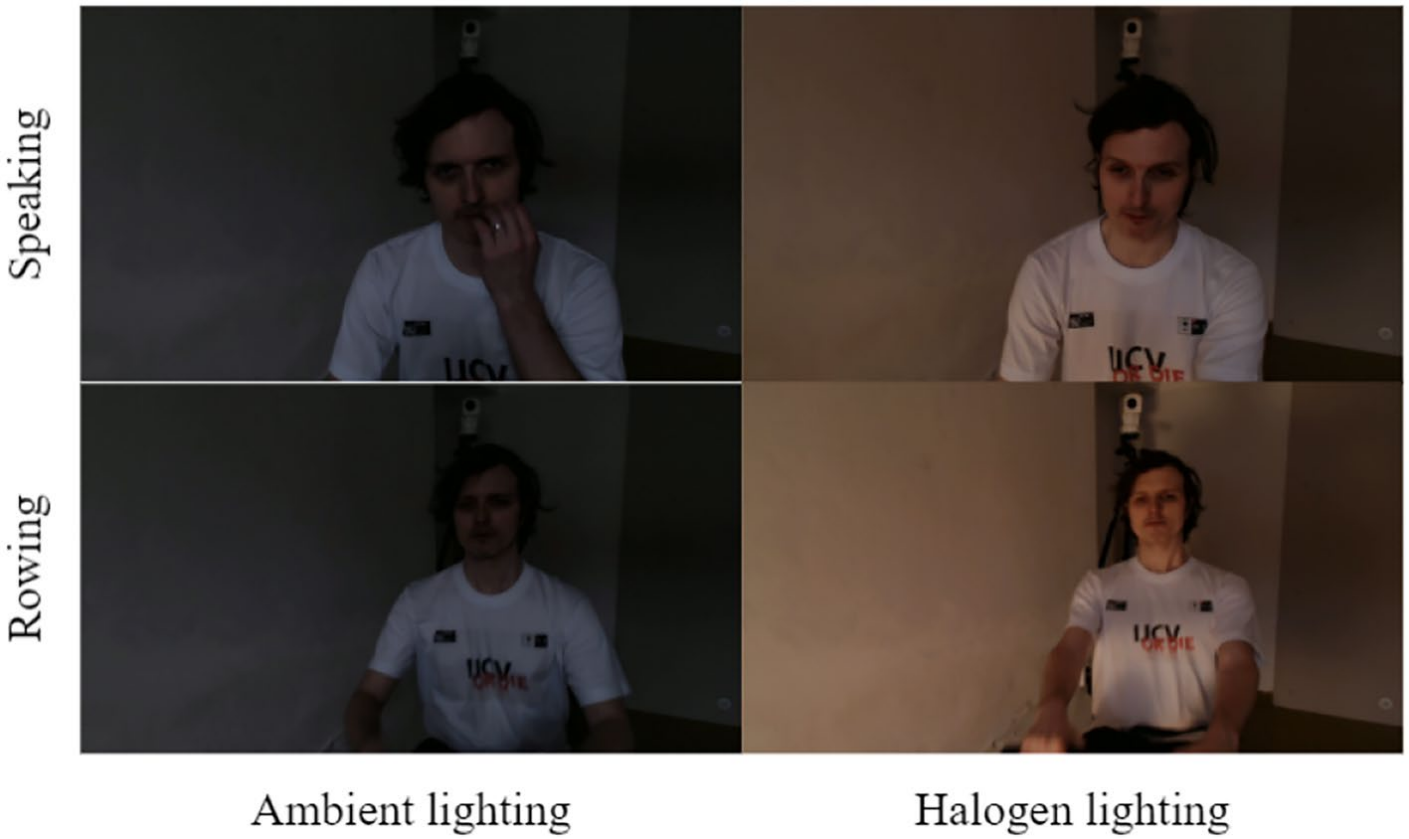
Captured images of one test subject.

An ECG fitness dataset provided by Czech Technical University^5^ was used for the experiment. We chose this dataset for three reasons: First, this dataset was organized with videos of 17 subjects (14 men and 3 women) performing physical activities on fitness machines^5^. They performed four movements: speaking, rowing, exercising on a stationary bike, and exercising on an elliptical trainer. In particular, the subjects performed the speaking and rowing operation twice, once in the presence of halogen light, resulting in a strong 50 Hz (100 Hz) temporal interference, and the other in its absence^5^. Second, the dataset was created using three lighting setups: natural light from a nearby window, 400 W halogen light, and 30 W LED light^5^. Third, because this dataset contains raw data, it can be processed and used as required for a predesigned experiment. Figure 4 shows a test subject under different lighting conditions and physical movements. In addition, 207 video images with a resolution of 1920 $\times$ 1080, 30 frames, and a 1-min video were saved in an uncompressed YUV 4:2:0 planar pixel format, depending on the subject, fitness actions performed by each subject, and environment^5^. Some videos were unavailable in the dataset. Additionally, the HR was measured according to the timestamp of each image.

### Data set preparation

**Fig. 5:**
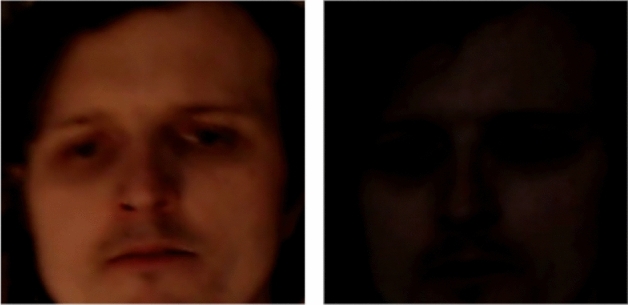
Cropped image samples.

We performed the following procedure to divide the ECG fitness dataset into training, validation, and test sets. First, we decomposed all 207 1-min video recordings on the ECG fitness dataset into a YUV file format video by one frame using the FFmpeg software. To extract face images from all YUV files decomposed one frame at a time, we extracted the coordinates where the face was located using a tool based on the SeetaFace2. We cropped all YUV files into 256 $\times$ 256 facial images using FFmpeg. Figure 5 shows the cropped image samples from the dataset. We divided the generated YUV facial image files into training, validation, and test sets at a ratio of 70:15:15. We classified the HR into eight image sets, and each cropped face image by HR in a certain section, as shown in Table 2. The HR section was divided into eight classes with seven beats/min (bpm) intervals between 83 and 124 bpm. Dataset labeling uses the estimated timestamp and HR, where the video is captured from the dataset. We prepared files in JPEG format as the test dataset. The PNG files were compressed into JPEG format using libjpeg-turbo. Subsequently, the JPEG quality factor (QF) was set differently to create a different type of test dataset. We set the JPEG QF to 10–90 in steps of 10, as shown in Table 3.

**Table 2:** Heart rate section and number of data.

Set index	Heart rate section (bpm)	Number of data		
		Training	Validation	Test
0	Under 82	43,481	9534	9196
1	83–89	22,028	4736	4497
2	90–96	31,720	6782	6963
3	97–103	34,988	7779	7310
4	104–110	30,741	6884	6047
5	111–117	31,553	6685	6784
6	118–124	34,509	6748	7885
7	Above 125	18,847	4058	4388
Total		247,867	53,206	53,070

**Table 3:** Compressed size of the test data.

JPEG QF	Total size (kB)	Compressed size compared to the raw YUV
10	127,611	2.5%
20	157,043	3.1%
30	182,137	3.6%
40	206,774	4.1%
50	231,747	4.5%
60	259,149	5.1%
70	302,871	5.9%
80	377,430	7.4%
90	560,184	11.0%

### Training environment

The development environments for our experiments were set up in two parts. Facial crops and datasets were prepared using Microsoft Windows 10 with Visual Studio C++ 2017. The classification part was developed under Ubuntu 16.04, using Python 3.6, Tensorflow-gpu 1.9.0, and Keras 2.2.0. The training processes were executed with a hardware configuration of Intel i7-7700K@4.2GHz, DDR4 32 GB RAM, Nvidia GeForce RTX-2070 Super 8 GB with CUDA 11.1. The test processes were executed with a hardware configuration of Intel i7-6700K@4.0GHz, DDR4 32 GB RAM, and Nvidia GeForce GTX-1660 Ti 6 GB with CUDA 10.1. We used a certain process to generate pre-trained models. Two images were used as the input batch. In the training process, we used a stochastic gradient descent with backpropagation. The training rate was set to 0.00001, with a momentum of 0.9. The models were trained up to 50 iterations. During these iterations, the mean square error function was used as the loss function to guide training.

### Training results of the NN models

Fifty iterations of the training and validation processes confirmed the stability of the weighted models and their performance, as presented in Table 4.

**Table 4:** Training and validation results.

Model	Training		Validation	
	Loss	Accuracy	Loss	Accuracy
DenseNet-121	0.2419	93.51%	0.7970	80.69%
VGG-16	0.0049	99.87%	0.1128	97.21%
Inception V3	0.0044	99.87%	0.1188	97.01%

The training and validation accuracies of DenseNet-121 are 93.51% and 80.69%, respectively. DenseNet-121 exhibited the lowest performance in terms of both the accuracy and loss. In addition, it exhibited the lowest performance among the three networks. VGG-16 exhibited the highest training and validation accuracies of 99.87% and 97.21%, respectively. While the training loss continued to decrease to 0.0049, the validation loss remained approximately 0.11. The accuracy of Inception V3 was 99.87% for training and 97.01% for validation. The training loss continued to decrease to 0.0044 and the validation loss reached 0.1188. VGG-16 and Inception V3 achieved the highest training accuracy. VGG-16 exhibited the highest validation accuracy and lowest loss compared with Inception V3 and DenseNet-121. Furthermore, approximately 40–50 training iterations could limit the accuracy.

### Cross validation

To identify problems such as data selection bias or data distribution bias, we use a 5-fold cross validation^21^ process to examine the training results. We used the training and validation data from the previous step as a database. Then, these images in the database were randomly split into five folds for cross-validation. The image numbers for each fold are listed in Table 5.

**Table 5:** 5-Fold cross validation data.

k-th fold	Number of data	
	Training	Validation
1	240,517	60,572
2	240,693	60,396
3	240,877	60,212
4	241,044	60,045
5	241,201	59,888

We collected the results for each fold of each model after 50 iterations. The results in Table 6 show that the average classification accuracy and loss for each model have no significant differences for each fold. However, Desenet-121 showed better performance in terms of validation accuracy than training accuracy.

**Table 6:** 5-Fold cross validation results.

Model	Training		Validation	
	Loss	Accuracy	Loss	Accuracy
DenseNet-121	0.32826	89.28%	0.3094	94.67%
VGG-16	0.00708	99.84%	0.17674	95.72%
Inception V3	0.00534	99.83%	0.15052	96.24%

### Test dataset results.

**Figure 6:**
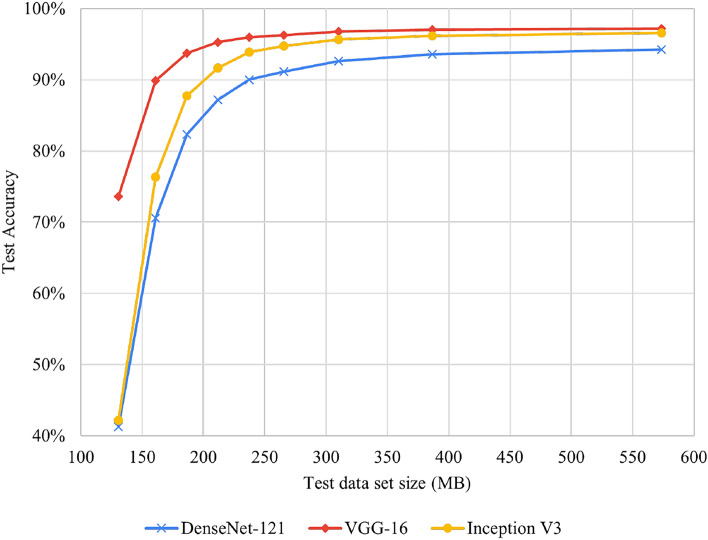
Test accuracy of three NN models.

We used compressed images with different QF values as inputs to evaluate the test performance. Subsequently, we performed image classification and calculated the accuracy for each labeled section. Table 7 shows the classification accuracy of each network for images with QF = 50. The total accuracies of DenseNet-121, VGG-16, and Inception V3 are 90.01%, 95.95%, and 93.88%, respectively. Subsequently, we calculated the total accuracy of each NN model at different QF values. Figure 6 presents the test accuracy results for DenseNet-121, VGG-16, and Inception V3 for different QFs. The x axis represents the total size of the test data. The highest accuracies for DenseNet-121, VGG-16, and Inception V3 were 94.23%, 97.20%, and 96.58%, respectively, whereas QF = 90. Consequently, all the models exhibited accuracies above 90% when the QF value was above 50. The differences between the highest and lowest accuracies in the QF range are 4.22%, 1.25%, and 2.69%, respectively.

**Table 7:** Test accuracy on compressed test data.

JPEG QF	Compressed ratio	DenseNet-121	VGG-16	Inception V3
10	2.50%	41.25%	73.59%	42.18%
20	3.10%	70.57%	89.88%	76.36%
30	3.60%	82.31%	93.73%	87.76%
40	4.10%	87.21%	95.28%	91.63%
50	4.50%	90.01%	95.95%	93.88%
60	5.10%	91.16%	96.29%	94.74%
70	5.90%	92.62%	96.78%	95.64%
80	7.40%	93.60%	97.05%	96.17%
90	11.00%	94.23%	97.20%	96.58%

VGG-16 not only showed the highest performance for high-quality images but also demonstrated an accuracy of 89.88% at QF = 20. The compressed size was only 3.1% of that of the raw facial-cropped image.

### Results analysis and discussion

From Tables 8, 9 and 10, we also analyzed the accuracy of the classification executed by each model with QF 50 images in the confusion matrices. The results show that the trained network model not only outputs a high accuracy for correct labeling but also shows a normal distribution on the nearest label class. This means that even if the image has been incorrectly labeled, the labeled heart rate has a larger chance of being detected with a difference of ± 7 bpm. The accuracies of DenseNet-121 and Inception V3 decreased faster in the QF range of 10–50. At the lowest compression quality (QF = 10), none of the networks achieved an accuracy of greater than 50%. Therefore, with a well-trained network model, the effect of lossy compression on the classification is limited. Moreover, the regular encoding configuration of the JPEG images satisfied the requirements of the models.

**Table 8:** Result of classification Densenet-121 QF 50.

		InputLabel							
		0	1	2	3	4	5	6	7
Output label	0	8627	77	5	0	0	0	2	2
	1	384	4151	172	37	12	13	21	14
	2	72	162	6245	137	31	28	5	6
	3	77	82	413	6808	316	196	93	32
	4	16	4	66	202	5308	370	102	33
	5	9	5	16	39	211	5767	330	30
	6	9	10	33	51	82	282	6739	148
	7	2	6	13	36	87	128	593	4123
$Acc^1$		93.8	92.3	89.6	93.1	87.7	85.0	85.4	93.9

1. Accuracy in percentage (%).

**Table 9:** Result of classification VCG-16 QF 50.

		InputLabel							
		0	1	2	3	4	5	6	7
Output Label	0	9148	50	9	2	0	3	4	1
	1	42	4351	64	8	0	5	2	2
	2	3	70	6703	83	13	6	2	1
	3	1	11	142	7052	98	43	12	8
	4	2	5	22	116	5731	189	60	28
	5	0	6	17	19	119	6246	132	23
	6	0	3	5	18	58	253	7542	175
	7	0	1	1	11	28	39	131	4150
*Acc*		99.4	96.7	96.2	96.4	94.7	92.0	95.6	94.5

**Table 10:** Result of classification Inception V3 QF 50.

		InputLabel							
		0	1	2	3	4	5	6	7
Output label	0	9039	57	14	1	3	0	1	0
	1	95	4249	79	12	3	2	1	0
	2	5	103	6527	146	12	9	3	0
	3	4	30	240	6916	194	35	13	5
	4	21	17	55	141	5498	224	49	32
	5	20	16	25	49	220	6187	218	50
	6	7	23	11	27	84	276	7253	146
	7	5	2	12	18	33	51	347	4155
*Acc*		98.2	94.4	93.7	94.6	90.9	91.2	91.9	94.6

While these findings highlight the resilience of the evaluated models under compression, it is worth noting how this study complements prior work on heart rate estimation. For example, state-of-the-art approaches such as Špetlík et al. ^5^ use convolutional neural networks to estimate heart rate from facial videos, demonstrating strong performance even under challenging conditions. Their method was evaluated on the ECG-Fitness dataset, which contains raw, high-resolution, uncompressed videos of subjects performing physical exercise under varying lighting conditions and with significant motion, achieving an RMSE of 19.15 bpm. This highlights a focus on robustness to real-world acquisition challenges. In contrast, our method addresses the task of heart rate interval classification using bandwidth-efficient, heavily compressed image data. Despite aggressive JPEG compression, our approach achieves up to 97.20% accuracy at quality factor 90 using the VGG-16 backbone, indicating that reliable classification performance can be maintained even under compression–an insight not typically explored in regression-based state-of-the-art methods.

**Fig. 7:**
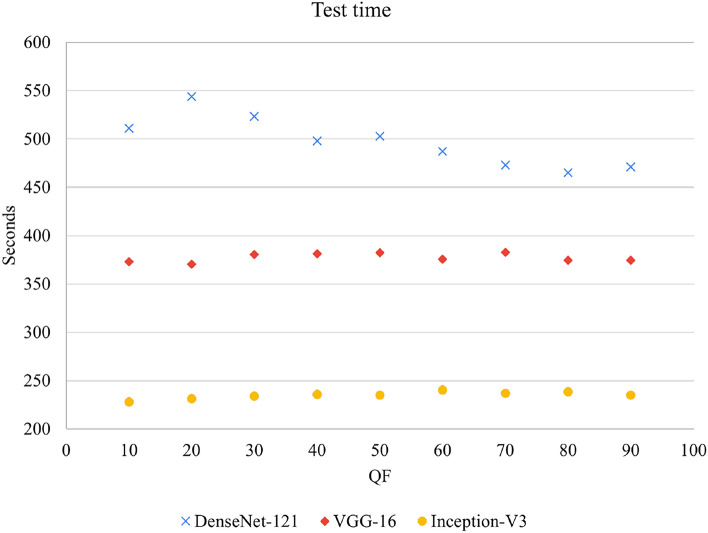
Time complexity of three NN models.

We also examined the time complexity of each model to process the test dataset. In Fig. 7, the geometric mean runtimes of these tests were 496.70, 377.17, and 234.94 seconds respectively. Inception-V3 had the fastest execution time, with a processing speed of 225 frames per second. In contrast, the speed of DenseNet-121 was 106 frames per second.

A limitation of our evaluation is that uncompressed raw datasets are rare in this study field. This limitation leads to the classification of heart rate intervals that is rough compared to other methods.

### Limitations and future work

Although the proposed method demonstrates high accuracy and efficiency in remote heart rate classification using compressed facial images, several limitations should be acknowledged. First, the experimental evaluation relied on a single dataset consisting of 17 subjects with a gender imbalance (14 males and 3 females). This relatively small and homogeneous sample size may limit the generalizability of the findings. Second, although various compression qualities were tested, only JPEG compression was considered, which may not fully represent real-world variations in compression artifacts across different codecs or formats.

Furthermore, the HR classification was based on discrete intervals rather than continuous estimation, which may lead to a loss in precision compared to regression-based methods. In addition, the current system assumes that facial detection and cropping are successfully performed, without addressing possible failures or noise in real-world deployment.

Future work will aim to incorporate larger and more diverse datasets to better evaluate the model’s robustness and generalizability. We also plan to explore other types of compression algorithms (e.g., HEVC, AVIF) and investigate the influence of color-space conversions. Finally, extending the model to support continuous heart rate regression and deploying it on edge devices are promising directions for enhancing its real-world applicability.

## Conclusion

Through the implementation of the system and reviewing the results, we observe that the NN-based remote HR classification method achieves good performance. Our evaluation confirmed that the VGG-16 yielded the highest HR classification performance. In addition, lossy image compression had a slight effect on HR accuracy. This result suggests that a system with an appropriate design and NN can obtain useful HR classification results under low-bitrate conditions. The proposed evaluation method provides a solution with a low computational complexity and bandwidth required for HR interval detection. Further studies are needed to investigate the effects of color-space conversion and other modern video codecs.

## Data Availability

All data generated or analysed during this study are included in this published article: Spetlik, R., Franc, V., Cech, J. and Matas, J. (2018) Visual Heart Rate Estimation with Convolutional Neural Network. In Proceedings of British Machine Vision Conference, 2018.
